# Efficient Removal of Ciprofloxacin from Contaminated Water via Polystyrene Anion Exchange Resin with Nanoconfined Zero-Valent Iron

**DOI:** 10.3390/nano13010116

**Published:** 2022-12-26

**Authors:** Yaqin Song, Ying Zeng, Ting Jiang, Jianqiu Chen, Qiong Du

**Affiliations:** School of Engineering, China Pharmaceutical University, Nanjing 211198, China

**Keywords:** ciprofloxacin, nZVI/PA nanocomposite, removal mechanism, transformation pathways

## Abstract

Ciprofloxacin (CIP), an important emerging contaminant, has been frequently detected in water, and its efficient removal has become an issue of great concern. In this study, a nanocomposite material nZVI/PA was synthesized by impregnating nanoscale zero-valent iron (nZVI) inside a millimeter-sized porous host (polystyrene-based anion exchange resin (PA)) for CIP removal. The nZVI/PA composite was characterized by field emission scanning electron microscopy coupled with energy-dispersive X-ray, transmission electron microscopy, X-ray diffraction, as well as X-ray photoelectron spectroscopy, and it was confirmed that nZVI was uniformly dispersed in PA with a small particle size. Furthermore, several key factors were investigated including initial solution pH, initial CIP concentration, co-existing ions, organic ligands, and dissolved oxygen. The experimental results indicated that the nZVI/PA composites exhibited a high removal efficiency for CIP under the conditions of initial pH 5.0, and initial CIP concentration 50 mg L^−1^ at 25 °C, with the maximum removal rate of CIP reaching 98.5%. Moreover, the nZVI/PA composites exhibited high efficiency even after five cycles. Furthermore, quenching tests and electron spin resonance (ESR) confirmed that CIP degradation was attributed to hydroxyl (·OH) and superoxide radicals ($\cdot O_{2}^{-}$). Finally, the main degradation products of CIP were analyzed, and degradation pathways including the hydroxylation of the quinolone ring, the cleavage of the piperazine ring, and defluorination were proposed. These results are valuable for evaluating the practical application of nZVI/PA composites for the removal of CIP and other fluoroquinolone antibiotics.

## 1. Introduction

Fluoroquinolone antibiotics are synthetic antibiotics with broad-spectrum antibacterial effects and are effective against pathogenic Gram-negative and Gram-positive bacteria [1,2]. Ciprofloxacin (CIP), an important fluoroquinolone antibiotic, is widely used to treat bacterial infections in human medicine, poultry livestock, and aquaculture [3,4]. However, after ingestion, 20–60% CIP can be excreted to surface water, ground water, and industrial wastewater [5,6] in its pharmacologically active form [7], and the concentration of detected CIP has ranged from ng L^−1^ to mg L^−1^ [8]. Moreover, the concentration of antibiotics in the medication factories has exceeded 50 mg L^−1^ [9,10]. Unfortunately, even a low concentration can have toxic effects on fauna and flora, with severe impacts on aquatic ecosystems, and can also produce antibiotic-resistant strains of bacteria [11]. CIP cannot be removed effectively by wastewater treatment plants due to its low biodegradability, and it has been proven to be one of the most persistent antibiotics in secondary wastewater treatments [12]. Therefore, CIP must be removed from water using different effective technologies.

Novel materials have captured increased attention for diverse uses, such as removing different pollutants [13,14,15,16]. Many studies have investigated the removal of antibiotics, such as ultrafiltration [17], nanofiltration [18], flocculation [19], coagulation [20], and adsorption [21]. However, these methods only enrich contaminants and transfer them from one phase to another. The contaminants are not degraded or mineralized and still have high costs for subsequent treatment. Advanced oxidation processes (AOPs) are attractive technologies to remove antibiotics from the environment, as they can degrade or mineralize organic contaminants into carbon dioxide, water, and inorganic ions. Previous reports have applied the following AOPs for CIP degradation: Fenton oxidation [22], Fenton-like oxidation [23], photocatalysis [24], ozonation oxidation [25], and catalytic oxidation [26]. As one of the classic AOPs, Fenton oxidation has been widely used to treat organic contaminants in sewage due to its high efficiency and environmental friendliness [27]. The traditional Fenton process is the reaction of ferrous ions (Fe^2+^) with hydrogen peroxide (H_2_O_2_) to produce hydroxyl radicals (·OH) and is a homogeneous reaction [28]. Nevertheless, the traditional Fenton process is only carried out in a limited pH range (2.0–4.0), and requires a large amount of ferrous salts, which could cause the loss of iron and forms a large amount of sludge, forming secondary pollution [29,30]. In order to solve this problem, heterogeneous Fenton-like oxidation technology has developed rapidly, with a focus on reusing materials.

Recently, with the introduction of nano zero valent iron (nZVI) as the source of Fe (II), the Fenton-like process had been further improved. Nanoscale zero-valent iron has been demonstrated to degrade antibiotics in Fenton systems [27]. Nevertheless, nZVI may trend to agglomerate into necklace-like structures due to its high energy and intrinsic magnetic interaction, which reduces the reactivity of the nZVI and results in poor mobility [31]. To date, significant attempts have been focused on the modification of aggregation alleviation of nZVI, such as silica [32], zeolite [33], activated carbon [31], sepiolite [34], biochar [35], and resin [36]. Porous polystyrene resin is a fascinating supporting material due to its large specific surface area, high porosity, low cost, easy regeneration, high stability, and specific modifiable group [37].

Hence, in this study, microporous polystyrene anion exchanger resin (PA)-supported nZVI (nZVI/PA) was synthesized for the degradation of CIP. The purposes of the present work were as follows: (1) to evaluate the removal efficiency of CIP in different factors by nZVI/PA; (2) to identify the main free radicals generated from the nZVI/PA system; (3) to explore the mechanisms and degradation pathways of CIP by nZVI/PA.

## 2. Materials and Methods

### 2.1. Materials

Ciprofloxacin hydrochloride (98%) was purchased from Aladdin Chemical Reagent Co., Ltd. (Shanghai, China). The commercial ZVI powder (500 mesh, particle size < 30 μm) used in this study was supplied by Suzhou Feixing Powder Metallurgy Co., Ltd. (Suzhou, China). The macroporous polystyrene anion exchange resin used in this study was purchased from HangZhou YongZhou Water Treatment Tech Co., Ltd. (Hangzhou, China). The PA was washed with NaCl solution, NaOH solution, HCl solution, and ethanol and then dried at 45 °C before use. All other chemicals were analytical grade and used without further purification. All solutions were prepared with pure water (EPED, Nanjing, China).

### 2.2. Synthesis of nZVI/PA Composites

The nZVI/PA composites were prepared by ion-exchange with ferric tetrachloride, followed by reduction with KBH_4_ using a liquid phase reduction method according to Reaction (1). The details are shown in our previous study [38]. In brief, 27.0 g FeCl_3_⋅6 H_2_O (1 M Fe^3+^) and NaCl (ample) were added into 100 mL solution (1 M HCl and 10% ethanol). Then, 1.0 g PA was added into the solution, which was shaken for 4 h. The nZVI nanoparticles were allowed to grow in situ inside PA after treatment with 7.2% KBH_4_, and was referred to as nZVI/PA.

(1) $${4\;\mathrm{Fe}}^{3+}{+3\;\mathrm{BH}}_{4}^{-}{+9\;H}_{2}O\to {4\;\mathrm{Fe}}^{0}{+3\;H}_{2}{\mathrm{BO}}_{3}^{-}{+12\;H}^{+}{+6\;H}_{2}$$

### 2.3. Removal of CIP

CIP stock solution (500 mg L^−1^) was prepared using pure water and stored at 4 °C before use. The batch experiments were carried out in a 150 mL Erlenmeyer flask in a water bath at 25 °C and 160 rpm. Firstly, 50 mg of fresh synthesized nZVI/PA composites was added to the Erlenmeyer flask containing 100 mL of CIP solution at an initial pH of 5.0 and concentration of 50 mg L^−1^. The effects of the initial pH, initial CIP concentration, co-existing ions, organic ligands, and dissolved oxygen (DO) were evaluated. In all experiments, the mixture system was maintained for 24 h, and then an aqueous solution was collected with a 5 mL syringe and filtered through a 0.22 mm nylon membrane filter for subsequent analysis. 

The removal kinetic experiments were performed by mixing a predetermined dosage of commercial ZVI (0.05 g L^−1^), PA (0.45 g L^−1^) and nZVI/PA (0.5 g L^−1^) with 500 mL of CIP solution (50 mg L^−1^) at pH 5.0, and without pH control during the reaction. At regular time intervals, the sample solution was collected with a 5 mL syringe and filtered through a 0.22 mm nylon membrane filter for subsequent analysis. In order to explore the effect of ROSs on the removal of CIP by the nZVI/PA, tert-butanol (TBA) and benzoquinone (BQ) were added before adjusting pH. For the reusability test, the nZVI/PA composites were regenerated after each test with 50% methanol and reduced by KBH_4_. All the results presented here are the average values of triplicate experimental results.

### 2.4. Analytical Methods

The concentration of CIP was carried out in a high-performance liquid chromatography (HPLC) system equipped with SPD-M20A detector at 280 nm, CTO-20AC column oven at 40 °C, SIL-20A HT automatic sampler, and two LC-20AT pumps (Shimadzu, Japan). The column of the HPLC system was a Waters Symmetry C18 column (4.6 × 150 mm, 5 mm). The mobile phases of two effluents were 74% H_2_O with 0.1% formic acid and 26% methanol (HPLC grade, TEDIA) at a flow rate of 1.0 mL min^−1^. The injection volume was 10 μL.

The degradation intermediates of CIP were analyzed using a liquid chromatography-mass spectrometry (LC-MS, Waters, Xevo G2-Xs QTof) with a positive electrospray mode over a mass range of 50–1500 m/z. LC was performed with a Waters ACQUITY UPLC BEH C18 column (2.1 × 100 mm, 1.7 mm). The mobile phase was the same as the HPLC system. Other parameters were set as follows: capillary voltage 2.5 kV, desolvation gas flow 30 mL min^−1^, cone gas flow 5 mL min^−1^, and desolvation temperature 300 °C.

Electron spin resonance (ESR, Bruker A320, Bruker, Karlsruhe, Germany) was used to identify the reactive oxygen species (ROSs) formed in the reaction. 5,5-Dimethyl-1-pyrroline N-oxide (DMPO) was used as the spin-trapping agent of ·OH and $\cdot O_{2}^{-}$. In this test, methanol was used to facilitate the $\cdot O_{2}^{-}$ detection because $\cdot O_{2}^{-}$ is sensitive to water. The initial pH of the reagents (methanol, water) were adjusted to 5.0 before the composites was added. The mixed solution was placed into a capillary tube containing 100 mM DMPO, which was then analyzed with an ESR spectrometer.

The total dissolved Fe was determined by an atomic adsorption spectrophotometer (AAS) (WFX-200, Rayleigh, Beijing, China).

## 3. Results and Discussion

### 3.1. Characterization of nZVI/PA Composites

**Figure 1:**
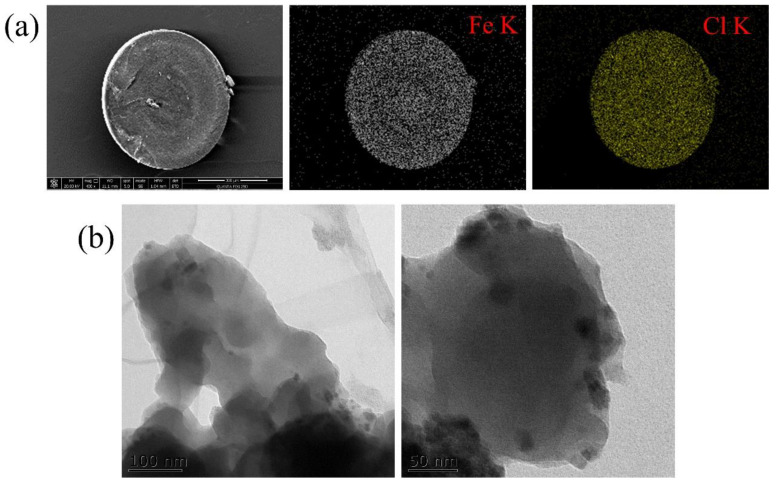
FESEM image of nZVI/PA composites with EDX spectra of elemental mapping in the cross section (**a**), and TEM images of nZVI/PA composites (**b**).

The morphology and the elemental distribution of nZVI/PA was characterized with field emission scanning electron microscopy coupled with an energy-dispersive X-ray (FESEM-EDX, QUANTA 400FEG, FEI, Oregon, USA) spectra. As shown in Figure 1a, the diameter of resin beads was 0.6–0.7 mm, and elemental Cl was homogeneously distributed in the cross section of nZVI/PA, which was the counterion Cl^−^ of PA. In addition, the elemental Fe was uniformly distributed in the whole matrix of PA. Furthermore, the uniform distributions reduced the particle size and the aggregation of the nanoparticles [39,40]. This was further demonstrated by transmission electron microscopy (TEM, JEM-2100F, JEOL, Tokyo, Japan). The TEM images of nZVI/PA showed that the nZVI was well dispersed as nanoparticles with sizes of 10–20 nm (Figure 1b). This was consistent with the report that nanoparticles were uniformly loaded into supporting materials [41].

**Figure 2:**
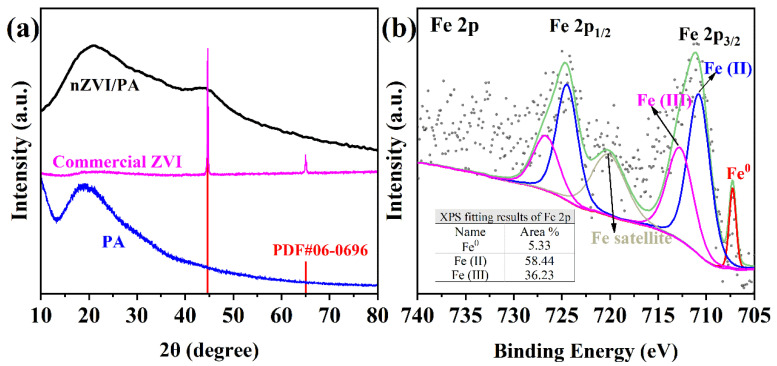
XRD patterns of PA, commercial ZVI, and nZVI/PA composites (**a**), and high-resolution XPS spectra of nZVI/PA composites (**b**).

The samples (PA, commercial ZVI and nZVI/PA) after milling were tested by X-ray diffraction (XRD, Ultimate IV, Rigaku, Japan) using Cu-Ka radiation (λ = 0.15418 nm) at a speed of 2°/min. As shown in Figure 2a, the wide and broad XRD pattern at a 2θ value of 20° indicated the amorphous form of PA, while the patterns of commercial ZVI at 2θ values of 44.7° and 56.0° corresponded to the (110) planes and (200) planes of Fe^0^ (JCPDS#06-0696), respectively. After the nZVI was loaded on PA, the broad pattern 2θ value of 44.7° revealed the presence of Fe0 and the crystallinity of composites was low. The XRD results showed that nZVI was successfully loaded into PA. To further verify the existence of Fe^0^ in the composites, X-ray photoelectron spectroscopy (XPS, EscaLab Xi+, ThermoFischer, Waltham, MA, USA) technology was used, and the results are shown in Figure 2b. The peak value at the binding energy of 707.0 eV was assigned to Fe^0^ and the peak values at the binding energies of 710.8 eV and 712.9 eV corresponded to Fe (II) and Fe (III) of Fe2p3/2, respectively. As shown in Figure 2b, because Fe^0^ on the surface of the composite was oxidized, only a relatively small amount of Fe^0^ (5.33%) existed. In addition, the different valence states of Fe indicated that nZVI has a core-shell structure with a core of Fe0 and a shell of iron oxide. The results were consistent with the previous literature [42,43], and XRD and XPS both showed that nZVI was successfully loaded into PA.

### 3.2. Effects of Key Factors on CIP Degradation by nZVI/PA

#### 3.2.1. Effect of Initial pH

**Figure 3:**
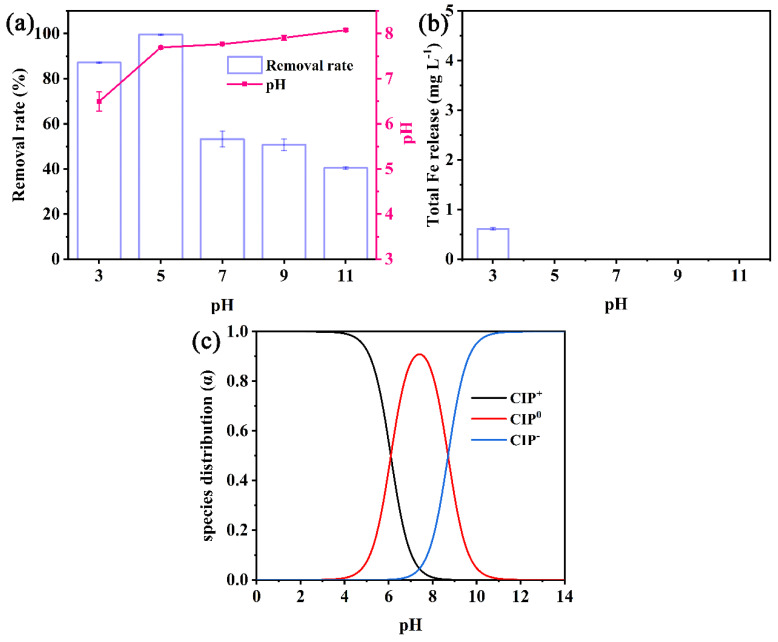
CIP removal efficiency and pH variation (**a**), total Fe release (**b**), and the species of CIP (**c**) in different pH by nZVI/PA composites.

Solution pH affects the species of CIP as well as the surface of the composites, which plays an important role in CIP removal. As shown in Figure 3a, the effects of different initial pHs on the removal of CIP by nZVI/PA composites were investigated. Figure 3a illustrates that acidic pH favored CIP degradation, with a maximum removal efficiency by nZVI/PA composites at PH = 5 of 98.5%; the removal efficiency significantly dropped to 40.4% with the initial pH further increasing to 11. The fast corrosion of nZVI to Fe^2+^ and the consequent further enhancement of the performance of the Fenton/Fenton-like oxidation under acidic conditions were the key factors for optimum removal efficiency of CIP. However, at a lower pH, the partial dissolution of nZVI was caused by corrosion (Figure 3b), which was not only easily weakens the adsorption of CIP onto nZVI/PA composites because of the partial dissolution of nZVI, but also excess H^+^ reacts with ·OH to generate H_2_O (Equation (2)). In contrast, under alkaline conditions, the inhibition in corrosion of nZVI as well as formation of a passivation film on the surface of nZVI could be the possible reason for decreased CIP removal capacity [44]. With pH increased to 5.0 and higher, the release of Fe was restrained, and the total Fe release was almost zero (Figure 3b). The result could be attributed to the immobilization of nZVI on PA, and demonstrates that nZVI/PA could reduce the loss of Fe and the production of Fe sludge in practical application.

(2) $$\cdot {\mathrm{OH}+H}^{+}{+e}^{-}\to H_{2}O$$

In addition, CIP is an amphoteric molecule, which has two acid-dissociation constants of 6.09 and 8.74 [45]. CIP has three different forms at different pH values: protonated (CIP^+^), non-protonated (CIP^0^), and deprotonated (CIP^−^), which are shown in Figure 3c. The final pH was lower than 8.74 after reaction (Figure 3a). The PA with the quaternary ammonium functional group had a slight adsorption capacity for CIP in the test pH 3.0–11.0.

The variation in the absorption band in the CIP aqueous solution with the wavelength range from 200 nm to 400 nm is depicted in Figure S1. A rapid decrease in the absorbance peak at 280 nm was observed in the reaction with the time increased, and the decrease of the absorption band suggests the removal of CIP by nZVI/PA.

#### 3.2.2. Effect of Initial CIP Concentration

**Figure 4:**
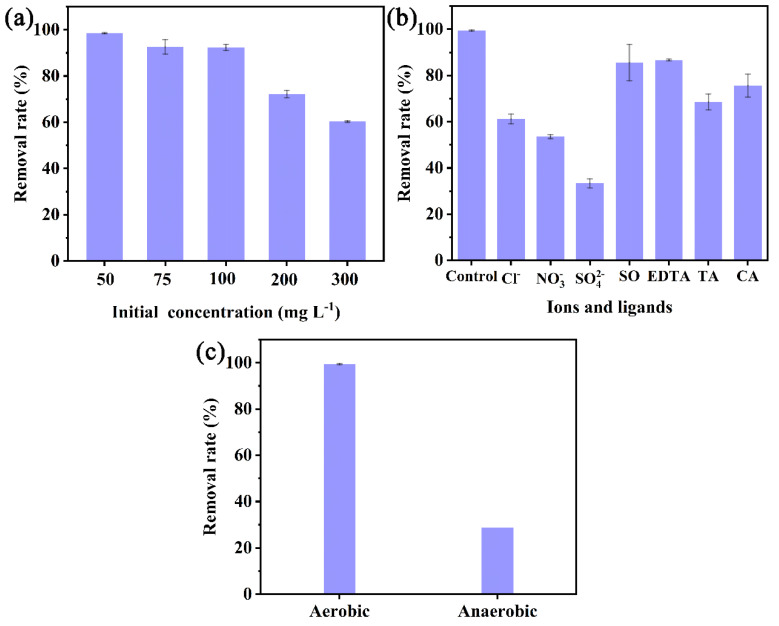
Effect of CIP initial concentration (**a**), co-existing ions and organic ligands (**b**), and DO (**c**) on CIP removal.

Although the concentration of CIP in the water environment is low, the concentration of wastewater discharged in some special places is very high. The concentration of livestock and aquaculture wastewater and pharmaceutical wastewater could reach tens of mg L^−1^ [10,46]. Based on published literature [47], concentrations up to 300 mg L^–1^ were identified as an extreme case. The performance of nZVI/PA for the removal of CIP with different concentrations (50−300 mg L^−1^) is shown in Figure 4a. It is significant that the degradation of CIP decreased with the increase of initial CIP concentration; 60.4% of CIP was removed in 24 h at the concentration of 300 mg L^−1^. The degradation of CIP by nZVI/PA was almost complete at low concentration (50−100 mg L^−1^), with degradation rates of 98.5%, 92.7%, and 92.3%, respectively. As the concentration increased to 200 mg L^−1^ and 300 mg L^−1^, the degradation rates decreased to 72.3% and 60.4%, respectively. The lower removal efficiency of CIP with the increase of concentration can be ascribed to the consumption of the limited radicals. Some byproducts, intermediates, and CIP competed for the limited reactive radicals with the increase of CIP concentration [48].

#### 3.2.3. Effect of Co-Existing Ions

Many investigations have reported that co-existing ions in wastewater have a significant influence on contaminant treatment [49,50]. Chloride ions (${\mathrm{Cl}}^{-}$), nitrate ions (${\mathrm{NO}}_{3}^{-}$), and sulfate ions (${\mathrm{SO}}_{4}^{2-}$) commonly exist in water environments, which can consume or further produce active species. The degradation of CIP by nZVI/PA with the addition of these co-existing ions was investigated. As shown in Figure 4b, the presence of these 2.0 mM ions had significant negative effects on CIP degradation by nZVI/PA. The inhibited effects of these ions followed the order of ${\mathrm{SO}}_{4}^{2-}$ > ${\mathrm{NO}}_{3}^{-}$ > ${\mathrm{Cl}}^{-}$ according to CIP degradation efficiency with the addition of these ions. As illustrated in Figure 4b, the degradation rates of CIP were 61.2%, 53.6%, and 33.4% in the presence of ${\mathrm{Cl}}^{-}$, ${\mathrm{NO}}_{3}^{-}$, and ${\mathrm{SO}}_{4}^{2-}$, respectively, with a decrease of 38.3%, 45.9%, and 66.1% compared to that without any ion addition, respectively. The inhibited effect of these inorganic ions (Cl^−^, ${\mathrm{NO}}_{3}^{-}$) was to quench ·OH through Equations (3) and (4) [51]. The newly formed species had less activity with contaminants compared with ·OH. In addition, the removal of CIP was completely masked due to the competition of coexisting ${\mathrm{SO}}_{4}^{2-}$, which was similar to Liu’s result [52].

(3) $$\cdot {\mathrm{OH}+\mathrm{Cl}}^{-}\to {\mathrm{ClOH}}^{\cdot -}$$

(4) $${\mathrm{NO}}_{3}^{-}+\cdot \mathrm{OH}\to {\mathrm{NO}}_{3}^{\cdot }\;$$

#### 3.2.4. Effect of organic ligands

The degradation of CIP by nZVI/PA with the addition of organic ligands, such as ethylenediaminetetraacetic acid (EDTA), potassium sodium tartrate (SS), sodium oxalate (SO), and sodium citrate (SC) were studied. As shown in Figure 4b, decreases of 12.8%, 30.9%, 13.9%, and 23.8% were detected with the addition of 0.1 mM EDTA, SS, SO, and SC ligands, respectively. These results can be ascribed to the organic ligands, which complexed with ${\mathrm{Fe}}^{2+}{/\mathrm{Fe}}^{3+}$ on the surface of iron, which decreased the availability of the active sites for CIP degradation. Moreover, organic ligands competed with CIP to consume reactive radicals, resulting in decreased CIP efficiency. Furthermore, the organic ligands were all anions and thus PA could adsorb them in the system.

#### 3.2.5. Effect of DO

Oxygen is one of the most important factors in a Fenton-like system. In this study, the anaerobic condition was investigated. The system was purged with nitrogen for 30 min. As shown in Figure 4c, the solution purged with nitrogen significantly decreased CIP removal. The removal efficiency was 28.8%, with a decrease of 70.7% in the absence of oxygen. These results could be ascribed to the purging nitrogen which led to the radical scavenging, and restraining the Fenton-like reaction. These results indicated that oxygen was necessary for the degradation of CIP. 

### 3.3. Reusability of nZVI/PA Composites

**Figure 5:**
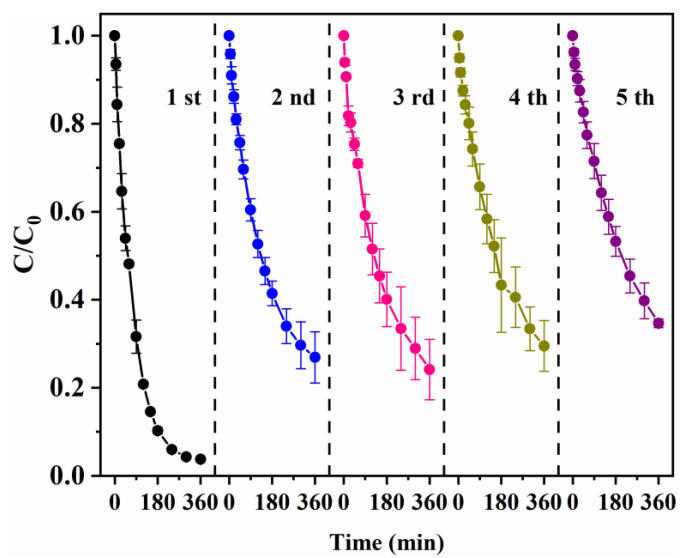
The recycling of nZVI/PA composites.

Regeneration and reuse to maintain the performance of materials is a key parameter from the perspective of potential application processing that is cost-effective [53,54,55]. The use of several cycles in the contaminant removal process without affecting its performance proves the commercial value of the composite [56,57]. Therefore, the reusability of nZVI/PA was tested through five cycles of degradation of CIP in this study, to maintain the cost-effectiveness of the process. Figure 5 demonstrates that the composites exhibited good reusability, as the CIP removal rate was 65.3% even after running five cycles. However, the CIP removal rate decreased as the regeneration times increased. This was mainly because the formation of iron oxides during the reaction impeded regeneration, and the procedures of experimental operation brought about material loss. The results show that nZVI/PA can be reused in multiple cycles without any significant loss of its performance, and it was a cost-effective material.

### 3.4. Comparison Study

**Figure 6:**
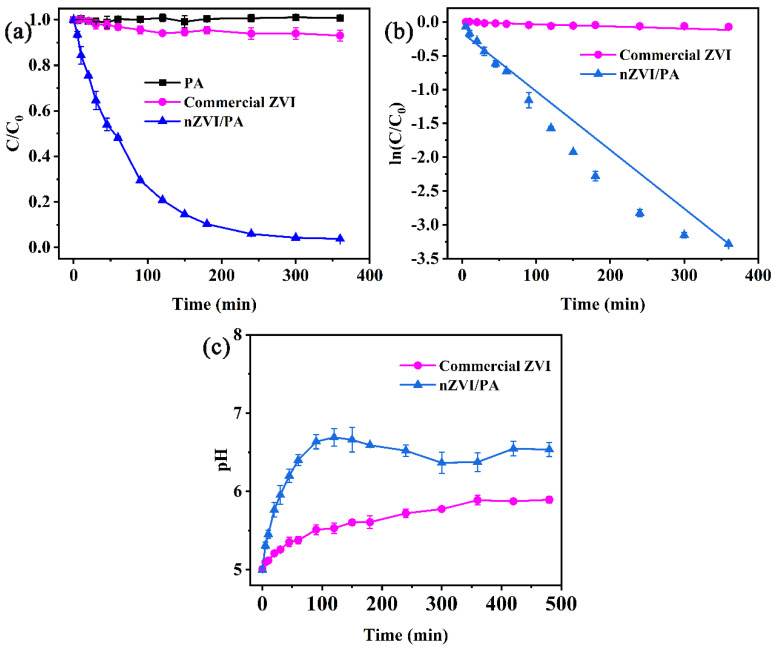
Time-dependent degradation (**a**), pseudo-first order kinetic curves (**b**), and pH variation (**c**) in the reaction of CIP by ZVI/PA.

The removal of CIP was compared among three materials, including the commercial ZVI, PA, and nZVI/PA, and the results are shown in Figure 6. As shown in Figure 6a, the removal of CIP was almost zero after 360 min by the host anion exchanger PA, suggesting that PA has no adsorption effect on CIP in pH 5.0. For the commercial ZVI, 8.2% removal of CIP was observed after 360 min, suggesting inefficiencies in the commercial ZVI. Compared with PA and commercial ZVI, the loaded nZVI showed a breakthrough for CIP degradation. nZVI/PA had the best removal efficiency reaching 96.2% within 360 min. The loaded iron could disperse the nZVI on PA, completely inhibit the agglomeration of nZVI, and accelerate the corrosion rate of nZVI, which increased the reactivity of the composites. The removal kinetics of SMX by commercial ZVI and nZVI/PA were fitted by a pseudo-first order kinetic model (Equation (5)), which is shown in Figure 6b. The kinetic fitting data are illustrated in Table S1. The removal kinetics agreed with the model after ZVI was supported on PA. The k_1_ value increased from 3.22 × 10^−4^ min^−1^ to 8.66 × 10^−3^ min^−1^ after ZVI was supported on PA. The removal rate for CIP degradation was enhanced by about 11.8-fold that of commercial ZVI. The pH variation of CIP removal is shown in Figure 6c; the pH values increased slowly from 5.0 to 5.9 and were stable at 5.9 in the commercial ZVI. However, the pH values increased to 7.0 in the first 90 min with the addition of nZVI/PA composites, and then dropped slowly to about 6.7. The increase of pH in both systems could be contributed to the corrosion of Fe through Equations (6) and (7) [52]. In addition, the obvious difference in pH indicated that the nZVI/PA composites had higher reactivity than the commercial ZVI.

The removal of CIP by nZVI/PA was compared with other materials reported in the literature, which are listed in Table 1. Compared with other Fe-based materials, nZVI/PA exhibited satisfactory removal capacity of CIP without the addition of other oxidants. Thus, nZVI/PA has excellent potential for practical application.

(5) $$\ln(C/C_{0})=-k_{1}.t$$

where *C*_0_ and *C* are the concentration of CIP at the beginning and after a period of time, respectively. *k*_1_ is the reaction rate constant of the pseudo-first order kinetic and t is the reaction time.

(6) $${2\mathrm{Fe}}^{0}{+O}_{2}{+2H}_{2}O\to {2\mathrm{Fe}}^{2+}{+4\mathrm{OH}}^{-}$$

(7) $${4\mathrm{Fe}}^{2+}{+O}_{2}{+2H}_{2}O\to 4{\mathrm{Fe}}^{3+}{+4\mathrm{OH}}^{-}$$

### 3.5. Mechanism of CIP Removal

#### 3.5.1. Identification and Detection of ROSs

**Figure 7:**
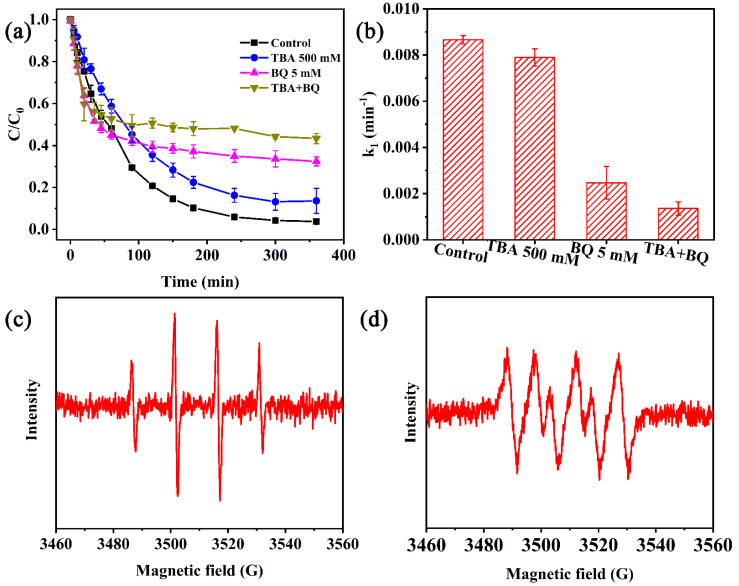
Inhibition effects of TBA and BQ (**a**), and *k*_1_ value of each scavenger (**b**) on the degradation of CIP, ESR spectra of DMPO spin trapping adducts of ·OH (**c**) and $\cdot O_{2}^{-}$ (**d**) in nZVI/PA.

Many previous studies have investigated the ROSs, such as ·OH and $\cdot O_{2}^{-}$, in Fenton-like systems [63,64]. To demonstrate the occurrence of ROSs in the nZVI/PA system, a series of chemical scavenging experiments were conducted. TBA is considered to be an efficient scavenger of ·OH due to its high reactivity with ·OH (3.8−7.6 × 10^8^ M^−1^ s^−1^), and BQ is considered an efficient scavenger of $\cdot O_{2}^{-}$ due to its high reactivity with $\cdot O_{2}^{-}$ (0.9−1.0 × 10^9^ M^−1^ s^−1^) [65,66]. TBA and BQ were added at concentrations of 500 mM and 5 mM, respectively, which corresponded to 3300: 1 and 33: 1 M ratios of probe compounds versus CIP, respectively. As shown in Figure 7a, with the addition of TBA, BQ, and TBA + BQ, the CIP degradation efficiencies were decreased to 86.4%, 67.6%, and 56.7%, respectively. Decreases of 9.8%, 28.6%, and 39.5%, respectively, were observed and the decrease of TBA + BQ was the sum of TBA and BQ. Correspondingly, the *k*_1_ value decreased from 0.0087 min^−1^ to 0.0079 min^−1^, 0.0025 min^−1^, and 0.0014 min^−1^ with the addition of TBA, BQ, and TBA + BQ (Figure 7b), respectively. The degradation efficiency reduced significantly with the quenching of ·OH and $\cdot O_{2}^{-}$, which revealed that ·OH and $\cdot O_{2}^{-}$ were the ROSs for the degradation of CIP by nZVI/PA. The generation of $\cdot O_{2}^{-}$ was formed through electrons (e^−^) reacting with oxygen. The successful cycle between ${\mathrm{Fe}}^{2+}$ and ${\mathrm{Fe}}^{3+}$ benefited the degradation of CIP, which was reported in previous studies [67].

To further validate the degradation of ROSs, ESR experiments were used to clarify the adducts between DMPO and radicals. As illustrated in Figure 7c, a specific spectrum with peak height ratio of 1:2:2:1 was detected, which represented the DMPO-·OH spin adducts [68]. Meanwhile, a special spectrum with an intensity ratio of 1:1:1:1 quartet signal was observed (Figure 7d), which represented $\mathrm{DMPO}-\cdot O_{2}^{-}$ [69]. The results revealed that both ·OH and $\cdot O_{2}^{-}$ were the ROSs in the degradation of CIP by nZVI/PA. The results are highly consistent with the results of chemical scavenger tests. 

#### 3.5.2. Reaction Mechanism

**Figure 8:**
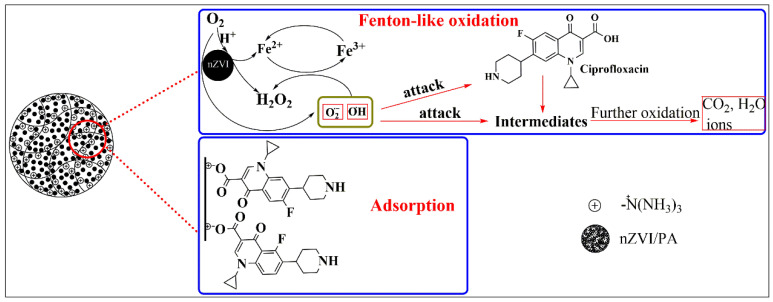
The reaction mechanism of CIP removal by nZVI/PA composites.

Based on the above and other results, a possible mechanism of nZVI/PA for CIP removal is proposed in Figure 8, which includes Fenton-like oxidation degradation and adsorption. For the oxidation process, firstly, nZVI supported on PA generated ·OH through Fenton-like reaction Equations (8) and (9) and gave a sustained supply of Fe^2+^ through Equation (10). In addition, electrons of nZVI rapidly consumed oxygen to form $\cdot O_{2}^{-}$ through Equations (11)–(13). Eventually, CIP was degraded by $\cdot \mathrm{OH}/\cdot O_{2}^{-}$ through Equation (14). For the adsorption process, the adsorption of CIP on composites was predominately as a result of sharing or exchanging the electrons between the active sites and CIP [70].

(8) $${\mathrm{Fe}}^{0}{+O}_{2}{+H}^{+}\to {\mathrm{Fe}}^{2+}{+H}_{2}O_{2}$$

(9) $${\mathrm{Fe}}^{2+}{+H}_{2}O_{2}\to {\mathrm{Fe}}^{3+}+\cdot {\mathrm{OH}+\mathrm{OH}}^{-}$$

(10) $${2\mathrm{Fe}}^{3+}{+\mathrm{Fe}}^{0}\to {3\mathrm{Fe}}^{2+}\;$$

(11) $${\mathrm{Fe}}^{0}\to {\mathrm{Fe}}^{2+}{+2e}^{-}$$

(12) $$e^{-}{+O}_{2}\to \cdot O_{2}^{-}$$

(13) $${\mathrm{Fe}}^{0}{+O}_{2}\to {\mathrm{Fe}}^{3+}+\cdot O_{2}^{-}\;$$

(14) $$\;\cdot O_{2}^{-}/\cdot \mathrm{OH}+\mathrm{CIP}\to {\mathrm{products}+\mathrm{CO}}_{2}{+H}_{2}O$$

### 3.6. Possible Degradation Pathways of CIP

**Figure 9:**
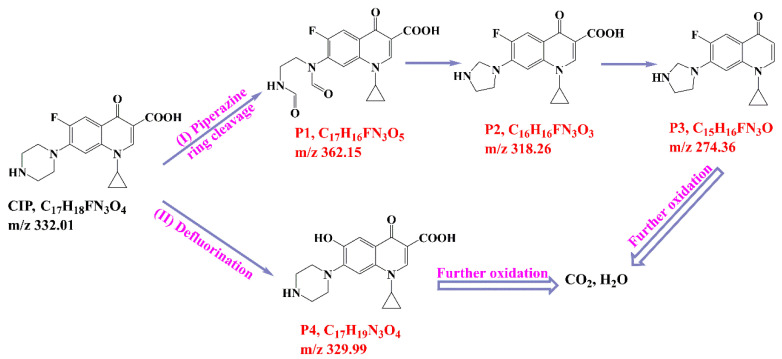
Possible degradation pathways for CIP by nZVI/PA.

To investigate the intermediates of CIP degradation and the degradation pathways of CIP by nZVI/PA composites, LC-MS was employed, and the degradation products at pH 5.0 were chosen to be investigated. The main products with m/z of 362, 318, 274, and 330 were detected and are summarized in Table 2 and in Figures S2 and S3. Based on the products, two possible degradation pathways were displayed in Figure 9. Pathway I was the stepwise oxidation degradation of the piperazine ring by ·OH and $\cdot O_{2}^{-}$, and pathway I was the hydroxylation of the quinolone ring by ·OH and $\cdot O_{2}^{-}$. The P1 was dialdehyde, which was produced by the breaking of the carbon–carbon bonds in the piperazine ring on the CIP molecule, which was then converted into imidazoline derivative P2 (m/z 318) via the loss of formaldehyde. Subsequently, the P2 by decarboxylation and loss of carbon dioxide led to the product P3 (m/z 274). Similar degradation products have been reported [71]. Pathway II is considered defluorination and hydroxylation to produce P4 (m/z 330). A similar degradation pathway was reported by Zhu et al. in hydroxyl radicals-based oxidation of CIP [66].

## 4. Conclusions

In this study, a nanocomposite material nZVI/PA was proposed to remove CIP from an aqueous system by immobilizing nZVI inside a millimeter-sized polymer. The performance of batch experiments under various operating parameters indicated that the optimal conditions were acidic (pH = 5.0) and aerobic conditions, with an initial CIP concentration of 50 mg L^−1^. There was little dissolution of Fe during use. Compared with commercial ZVI and PA alone, nZVI/PA possessed a higher removal efficiency for CIP, with the maximum removal rate of CIP reaching 98.5%. In addition, the reusability of nZVI/PA composites showed high removal efficiency even after five cycles. Chemical scavenging experiments and ESR revealed that ·OH and $\cdot O_{2}^{-}$ were the dominant reactive species responsible for CIP degradation. Furthermore, based on the LC-MS spectra, four degradation intermediates and two degradation pathways were proposed. Finally, this research indicates that nZVI/PA has great practical application potential for the removal of CIP and for other fluoroquinolone antibiotics from aqueous systems.

## Figures and Tables

**Table 1:** Comparison of CIP removal by nZVI/PA and other materials.

Materials	Initial pH	Initial Concentration (mg L^−1^)	Dosage (g L^−1^)	Removal Capacity (mg g^−1^)	Refs.
Wheat straw-nZVI	-	20	0.75	26.47	[58]
Pillared iron-UV-laponite	3.0	50	1.0	39.72	[59]
ZVI	2.5	22	2.5	7.04	[60]
Guar gum-nZVI	4.0	15	0.5	28.2	[61]
Biochar/nZVI/H_2_O_2_	3.0	100	0.4	154.75	[62]
nZVI/PA	5.0	50	0.5	96.2	This study

**Table 2:** Main products of CIP degradation detected by LC-MS.

m/z, [M+H]^+^	Molecular Weight	Molecular Formula	Molecular Structure	Refs.
332.04	331.35	C_17_H_18_FN_3_O_3_	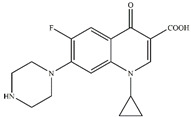	
362.15	361.33	C_17_H_16_FN_3_O_5_	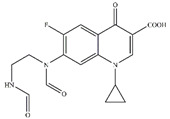	[66]
318.26	317.22	C_16_H_16_FN_3_O_3_	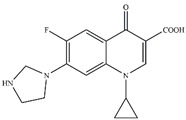	[72]
274.36	273.31	C_15_H_16_FN_3_O	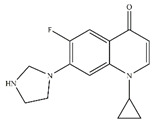	[71]
329.99	329.36	C_17_H_19_FN_3_O_4_	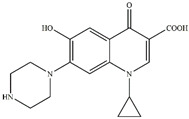	[73]

## Data Availability

Data can be made available upon request from the authors.

## Supplementary Materials

The following supporting information can be downloaded at: https://www.mdpi.com/article/10.3390/nano13010116/s1, Table S1. Kinetic parameters calculated by a pseudo-first order model. Figure S1. Absorption variation spectra of CIP aqueous solution in the initial pH 5.0 analysis. Figure S2. Mass spectrogram of CIP. Figure S3. Mass spectrogram of CIP degradation by nZVI/PA.
