# Supplementary material for: Efficient Removal of Ciprofloxacin from Contaminated Water via Polystyrene Anion Exchange Resin with Nanoconfined Zero-Valent Iron
Source: Nanomaterials (Basel). 2022 Dec 26;13(1):116. doi: 10.3390/nano13010116 (PMC9823821; doi:10.3390/nano13010116)
Supplement: Supplementary file 1 [file nanomaterials-13-00116-s001.zip › nanomaterials-2106107-supplementary.pdf]

Efficient Removal of Ciprofloxacin from Contaminated Water via Polystyrene Anion Exchange Resin with Nanoconfined Zero-Valent Iron

Yaqin Song, Ying Zeng, Ting Jiang, Jianqiu Chen * and Qiong Du *

School of Engineering, China Pharmaceutical University,
Nanjing 211198, China
* Correspondence: cjqer@163.com (J.C.); duqiong116@163.com (Q.D.);
Tel.: +86-25-8618-5190 (J.C.)

Table captions:

Table S1. Kinetic parameter calculated by pseudo-first order model.

Figure captions:

Figure S1. Absorption variation spectra of CIP aqueous solution in the initial pH 5.0.

Figure S2. Mass spectrogram of CIP.

Figure S3. Mass spectrogram of CIP degradation by nZVI/PA.

Table S1 Kinetic parameter calculated by pseudo-first order model.

Commercial ZVI dosage (g L^{-1})	nZVI/PA dosage (g L^{-1})	CIP concentration (mg L^{-1})	pH	k_1 (min^{-1})	R^2
0.05	-	50	5.0	3.22×10^{-4}	0.7517
-	0.5	50	5.0	8.66×10^{-3}	0.9947

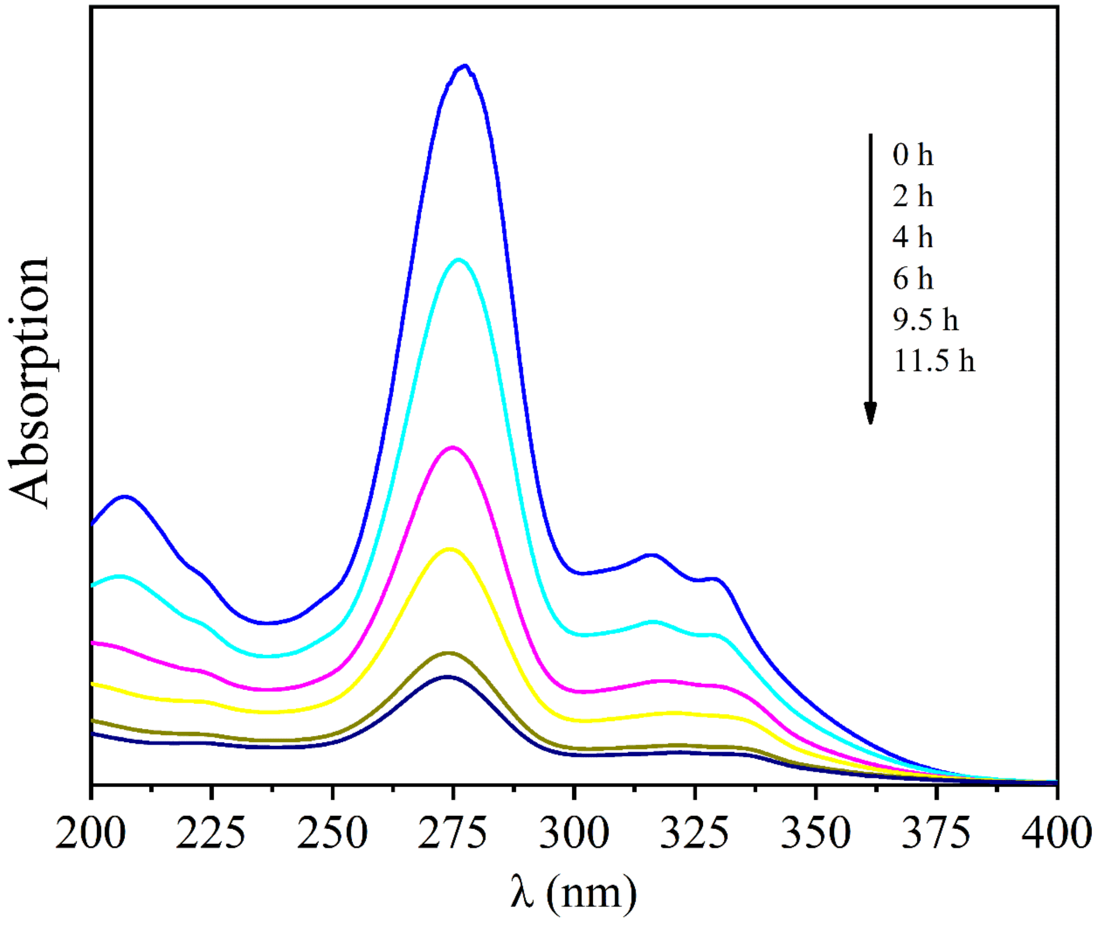

Figure S1. Absorption variation spectra of CIP aqueous solution in the initial pH 5.0.

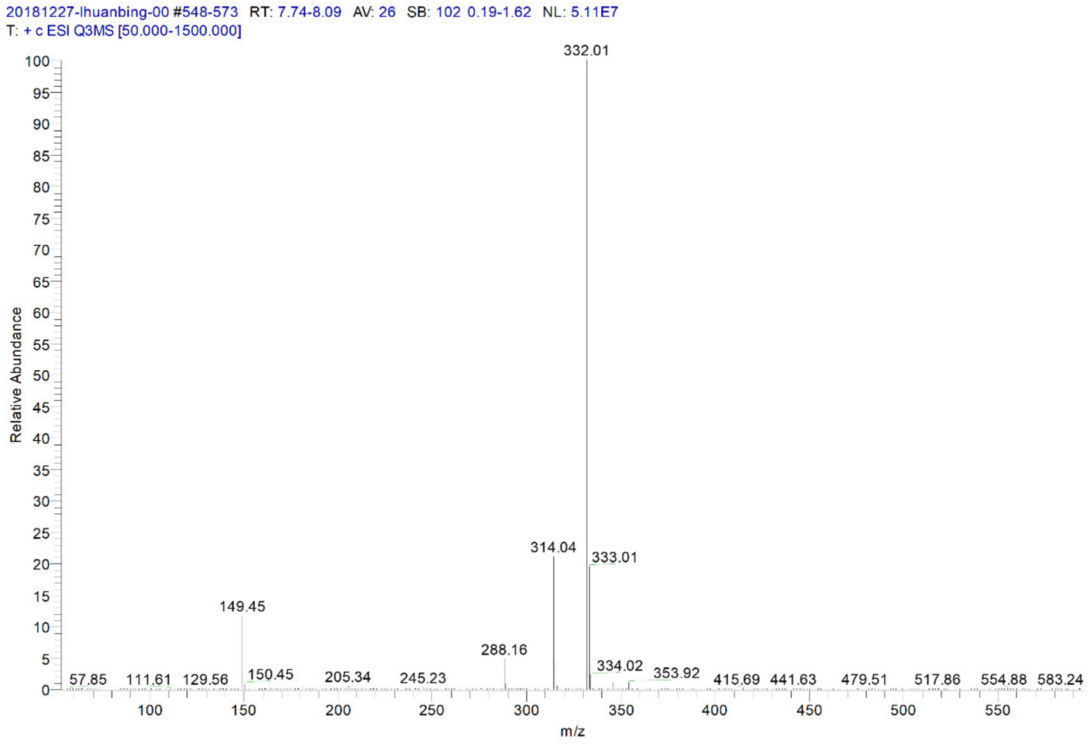

Figure S2. Mass spectrogram of CIP.

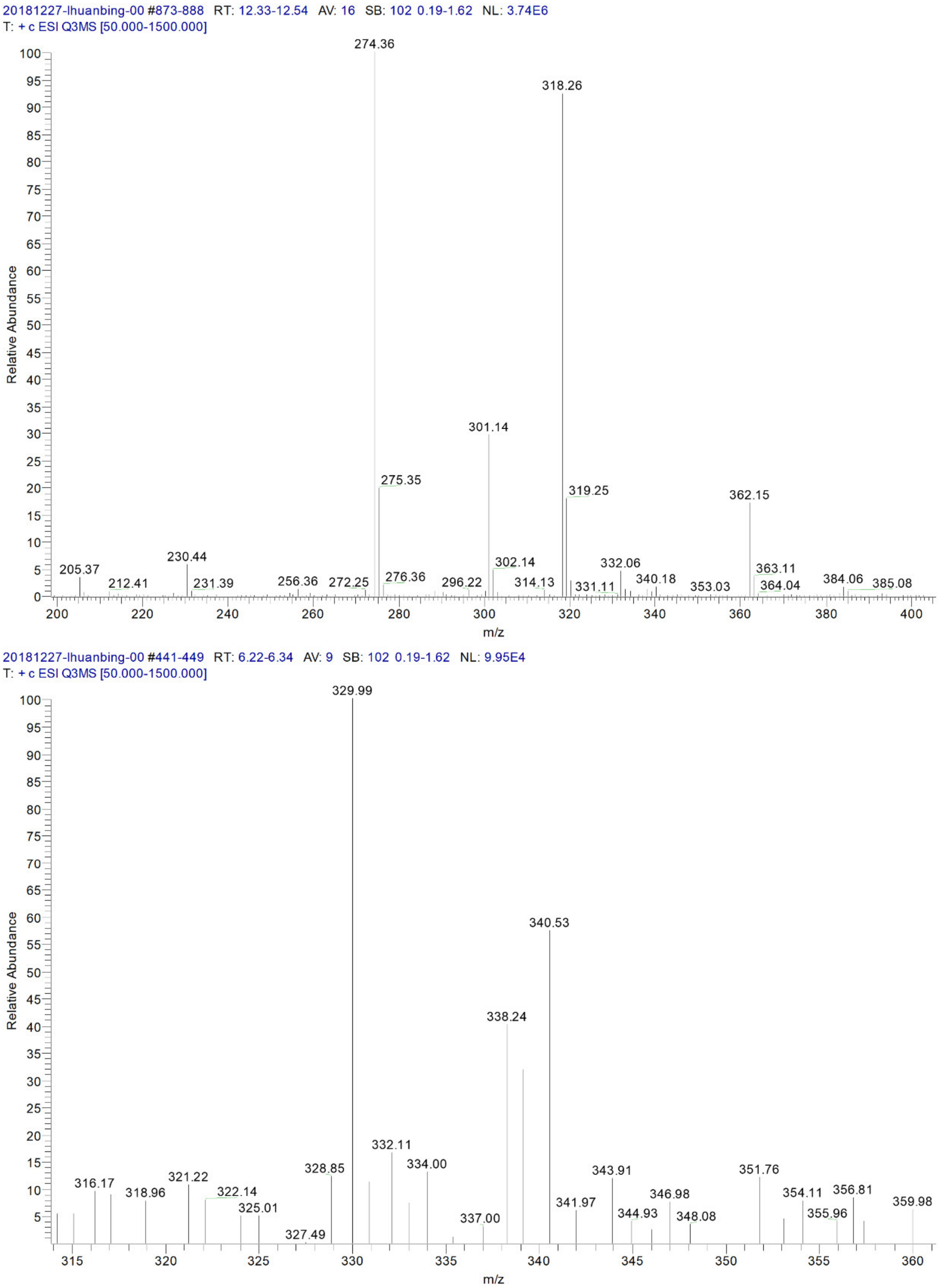

Figure S3. Mass spectrogram of CIP degradation by nZVI/PA.
